# Dopaminergic Neurons Respond to Iron-Induced Oxidative Stress by Modulating Lipid Acylation and Deacylation Cycles

**DOI:** 10.1371/journal.pone.0130726

**Published:** 2015-06-15

**Authors:** Sofía Sánchez Campos, Guadalupe Rodríguez Diez, Gerardo Martín Oresti, Gabriela Alejandra Salvador

**Affiliations:** Instituto de Investigaciones Bioquímicas de Bahía Blanca (INIBIBB), Universidad Nacional del Sur (UNS) and Consejo Nacional de Investigaciones Científicas y Técnicas (CONICET), Bahía Blanca, Argentina; Indian Institute of Integrative Medicine, INDIA

## Abstract

Metal-imbalance has been reported as a contributor factor for the degeneration of dopaminergic neurons in Parkinson Disease (PD). Specifically, iron (Fe)-overload and copper (Cu) mis-compartmentalization have been reported to be involved in the injury of dopaminergic neurons in this pathology. The aim of this work was to characterize the mechanisms of membrane repair by studying lipid acylation and deacylation reactions and their role in oxidative injury in N27 dopaminergic neurons exposed to Fe-overload and Cu-supplementation. N27 dopaminergic neurons incubated with Fe (1mM) for 24 hs displayed increased levels of reactive oxygen species (ROS), lipid peroxidation and elevated plasma membrane permeability. Cu-supplemented neurons (10, 50 μM) showed no evidence of oxidative stress markers. A different lipid acylation profile was observed in N27 neurons pre-labeled with [^3^H] arachidonic acid (AA) or [^3^H] oleic acid (OA). In Fe-exposed neurons, AA uptake was increased in triacylglycerols (TAG) whereas its incorporation into the phospholipid (PL) fraction was diminished. TAG content was 40% higher in Fe-exposed neurons than in controls. This increase was accompanied by the appearance of Nile red positive lipid bodies. Contrariwise, OA incorporation increased in the PL fractions and showed no changes in TAG. Lipid acylation profile in Cu-supplemented neurons showed AA accumulation into phosphatidylserine and no changes in TAG. The inhibition of deacylation/acylation reactions prompted an increase in oxidative stress markers and mitochondrial dysfunction in Fe-overloaded neurons. These findings provide evidence about the participation of lipid acylation mechanisms against Fe-induced oxidative injury and postulate that dopaminergic neurons cleverly preserve AA in TAG in response to oxidative stress.

## Introduction

Parkinson disease (PD) is a neurodegenerative disorder associated with progressive neurodegeneration, and it is characterized by pathological α-synuclein (α-syn) aggregation, oxidative stress, elevated tissue iron, and mis-compartmentalization of copper. The ethiopathogenesis of PD is centered in the progressive loss of dopaminergic neurons, however, the mechanism underlying this process is still poorly understood [1–4].

The central role of iron (Fe) in PD pathology is supported by reports showing that the administration of Fe chelators and the overexpression of iron-binding proteins are neuroprotective in PD animal models [5–7]. Higher levels of divalent metal transporter DMT1 and loss of DMT1 down regulation in response to increased levels of Fe have been observed in *post-mortem* brains from PD patients [8].

Even though Fe-overload is a well known event in PD, a complete characterization of the state of Cu metabolism is still missing. In this respect, an important number of reports described the decrease of this metal ion in the *substancia nigra* neurons of PD patients [9,10]. Lack of appropriate Cu concentrations could lead to disturbances in CuZn superoxide dismutase (SOD 1) expression and, in consequence, to an increased susceptibility to α-syn oligomerization and the generation of oxidative stress [9]. Therefore, during PD, Fe-overload and Cu deficiency provokes a surrounding environment with high levels of oxidative stress for dopaminergic neurons.

One decisive event for the survival of neuronal cells under oxidative stress injury is the maintenance of cellular membrane properties. The preservation of physicochemical membrane properties (fluidity and permeability) during oxidative stress events strongly depends on phospholipid (PL) remodeling and it is therefore related to cellular mechanisms for the regulation of fatty acids (FA) availability and storage.

Phospholipids are important structural and functional components of biological membranes, which behave as reservoirs of important lipid mediators. The acyl groups of PL are highly diverse, depending on the polar head group, and are distributed in an asymmetric manner [11,12]. Rapid turnover of the sn-2 acyl moiety of phospholipids is attributed to the concerted and coordinated actions of phospholipases A_2_s (PLA_2_s) and lysophospholipid acyltransferases (LPATs). Another important storage of polyunsaturated fatty acids (PUFA), such as arachidonic acid (AA) and docosahexaenoic acid (DHA), are triacylglycerols (TAG). TAG remodeling and their fatty acid composition are also crucial for the maintenance of cellular PUFA levels, a process catalyzed by diacylglycerol acyltransferases (DGAT). Storage of TAG occurs in lipid droplets (LD) that are considered dynamic organelles regulated in response to the metabolic cellular state [13–15].

A link between α-syn, lipid metabolism, fatty acid oxidation, and mitochondrial damage has been established in PD. It has been proposed that α-syn can interact with FA, oxidized lipids, and LD [16–18]. However, little is known about the mechanisms of FA turnover and lipid remodeling that operate during oxidative stress injury in dopaminergic neurons.

In view of the above, the purpose of the present work was to determine the role of lipid acylation mechanisms during Fe-induced injury in dopaminergic neurons and to study the effect of Cu supplementation during Fe-overload. To this end, N27 dopaminergic cells were incubated under Fe-overload and Cu-supplementation conditions and the state of lipid acylation/deacylation processes and their role in the neuronal response to oxidative injury were studied.

## Materials and Methods

### Materials

[^3^H] oleic acid (OA) and [^3^H] arachidonic acid (AA) were obtained from New England Nuclear-Dupont (Boston, MA, USA). Triton X-100, 3-(4,5-dimethylthiazol-2-yl)-2,5 diphenyltetrazolium bromide (MTT) and thiobarbituric acid (TBA) were obtained from Sigma-Aldrich (St. Louis, MO, USA). DCDCDHF was obtained from Molecular Probes (Eugene, Oreg., USA). Ferrous sulfate (J.T.Baker, cat.# 2070–01) was purchased from EMD Millipore (Millipore, Bedford, MA). Cupric sulfate (Mallinckrodt Chemicals) was purchased from Cientifica Nacional (Cientifica Nacional, Argentina).

All the other chemicals used in the present study were of the highest purity available. Antibodies: Goat polyclonal anti-LPAAT-θ(T-17) [sc 66789], rabbit polyclonal anti-DGAT1 (H-255) [sc 32861], mouse monoclonal anti-fatty acid synthase (FAS) (G-11) [sc48357], goat polyclonal anti-actin (C-11) [sc 1615], polyclonal horseradish peroxidase (HRP)-conjugated goat anti-rabbit IgG, polyclonal HRP-conjugated goat anti-mouse IgG and polyclonal HRP-conjugated bovine anti-goat IgG were purchased from Santa Cruz Biotechnology, Inc. (Santa Cruz, CA, USA). Acylation inhibitor (CI-976) and PLA_2_ inhibitors [arachidonoyl trifluoromethyl ketone (ATK), bromoenol lactone (BEL) and YM 26734] were purchased from Santa Cruz Biotechnology, Inc. (Santa Cruz, CA, USA). Polyvinylidene difluoride membranes were obtained from Millipore (Bedford, MA). Nile Red and Hoechst were from Molecular Probes. LDH-P UV AA kit was kindly provided by Wiener lab Group. TG color GPO/PAP AA and Colestat enzimático were purchased from Wiener lab Group.

### Cell culture

N27 cells, a rat mesencephalic dopaminergic cell line, were kindly donated by Dr. Patricia Oteiza (Department of Nutrition, UC DAVIS). Cells were grown and treated in RPMI 1640 medium supplemented with 10% (v/v) fetal bovine serum (FBS, Natocor, Argentina), 100 U/mL penicillin, 100 μg/mL streptomycin and 0.25 μg/mL amphotericin B at 37°C under 5% CO_2_.

### Experimental treatments

In all the experiments carried out, cells were plated in 35 x 10 mm Cell Culture Dishes and grown to 80%-90% confluence. Fe^2+^ and Cu^2+^ treatments were carried out in serum-free medium. Treatments with inhibitors were performed as follows: medium was removed and replaced by serum-free media. Inhibitors were subsequently added to the desired final concentration -10 μM BEL, 50 μM ATK, 10 μM Ym 26734 and 7,5 μM CI-976-; controls received vehicle alone (DMSO 0.05%). After 30 min, 1 mM Fe^2+^, 10 or 50 μM Cu^2+^, their combination or its vehicle (ultrapure water) were added and cells were incubated under these conditions for 24 h unless otherwise specified.

### Assessment of cell viability

Cell viability was assessed by MTT reduction assay. MTT is a water-soluble tetrazolium salt that is reduced by metabolically viable cells to a colored, water insoluble formazan salt. After treatments, MTT (5mg/ml) was added to the cell culture medium at a final concentration of 0.5 mg/ml. After incubating the plates for 2 h at 37°C in a 5% CO_2_ atmosphere, the assay was stopped and the MTT-containing medium was replaced with solubilization buffer (20% SDS, pH 4.7). The extent of MTT reduction was measured spectrophotometrically at 570 nm [19]. Results are expressed as a percentage of the control.

### Determination of lipid peroxidation

Lipid peroxidation was determined by the thiobarbituric acid reactive substances (TBARS) assay, which involves derivatization of malondialdehyde with thiobarbituric acid to produce a pink product that is quantified in a UV-VIS spectrophotometer. Briefly, after treatments, cells were scraped off into 300 μl of ice-cold water and mixed with 0.5 ml of 30% trichloroacetic acid. Then, 50 μl of 5 N HCl and 0.5 ml of 0.75% thiobarbituric acid were added. Tubes were capped, the mixtures were heated at 100°C for 30 min in a boiling water bath and the samples were centrifuged at 1000 x g for 10 min. TBARS were measured spectrophotometrically in the supernatant at 532 nm [20]. Results are expressed as a percentage of the control.

### Determination of cell oxidant levels

Cell oxidative stress was evaluated using the probe 5 (or 6)-carboxy-2’7’-dichlorodihydrofluorescein diacetate (DCDCDHF). This probe can cross the membrane and after oxidation, it is converted into a fluorescent compound. After the corresponding treatments, the medium was discarded, and RPMI medium containing 10 μM DCDCDHF was added. After 30 min of incubation at 37°C, the medium was removed, cells were rinsed three times with phosphate buffer saline (PBS) and they were subsequently either imaged with an epifluorescence microscope or lysed in a buffer containing PBS and 1% NP-40. Fluorescence in the lysates (λex = 495, λem = 530) was measured in a SLM model 4800 fluorimeter (SLM Instruments, Urbana, IL) [21]. Results are expressed as a percentage of the control.

### Measurement of LDH leakage

Lactate Dehydrogenase (LDH) leakage was determined as previously described [22] with slight modifications. After treatments, incubation medium was centrifuged at 1000 x g for 10 min at 4°C. The resulting supernatant was used to determine LDH activity, which was measured spectrophotometrically using LDH-P UV AA kit (Wiener lab.), following the manufacturer’s instructions. Briefly, the conversion rate of reduced nicotinamide adenine dinucleotide to oxidized nicotinamide adenine dinucleotide was followed at 340 nm. Results are expressed as percentage of the control.

### Evaluation of Mitochondrial Membrane Potential

To assess preservation of mitochondrial membrane potential, cultures were incubated for 20 min before fixation with the fluorescent probe MitoTracker Red (0.1 μg/mL), which labels mitochondria retaining their membrane potential with a bright red fluorescence.

### Western blot analysis

For the preparation of total cell extracts, cells (10 × 10^6^ cells) were rinsed with PBS, scraped and centrifuged. The pellet was rinsed with PBS and resuspended in 80 μl of a buffer containing 50 mM Tris, pH 7.5, 150 mM NaCl, 0.1% Triton X-100, 1% NP-40, 2 mM EDTA, 2 mM EGTA, 50 mM NaF, 2 mM β-glycerophosphate, 1 mM Na_3_VO_4_, 10 μg/ml leupeptin, 5 μg/ml aprotinin, 1 μg/ml pepstatin, 0.5 mM PMSF, and 0.5 mM DTT. Samples were exposed to one cycle of freezing and thawing, incubated at 4°C for 60 min and centrifuged at 10000 x g for 20 min. The supernatant was decanted and the protein concentration was measured. Cell lysates containing 25–50 μg of protein were separated by reducing 7–12.5% polyacrylamide gel electrophoresis and electroblotted to polyvinylidene difluoride membranes. Molecular weight standards (Spectra Multicolor Broad Range Protein Ladder, Thermo Scientific) were run simultaneously. Membranes were blocked with 5% nonfat dry milk in TBS-T buffer (20 mM Tris-HCl [pH 7.4], 100 mM NaCl, and 0.1% [wt/vol] Tween 20) for 1 h at room temperature and subsequently incubated with primary antibodies (anti-LPAAT-θ(T-17): sc-66789; DGAT1(H-255): sc-32861 and Fatty Acid Synthase (G-11): sc-48357), washed three times with TBS-T and then exposed to the appropriate HRP-conjugated secondary antibody for 1 h at room temperature. Membranes were again washed three times with TBS-T and immunoreactive bands were detected by enhanced chemiluminescence (ECL; GE Healthcare Bio-Sciences, Buenos Aires, Argentina) using standard X-ray film (Kodak X-OMAT AR; GE Healthcare Bio-Sciences). Immunoreactive bands were quantified using image analysis software (Image J, a freely available application in the public domain for image analysis and processing, developed and maintained by Wayne Rasband at the Research Services Branch, National Institute of Mental Health).

### Lipid phosphorus measurement

Lipid phosphorus (lipid P) was determined in total lipid extracts using the chemicals and reactions described by Rouser and collaborators[23].

### [^3^H] Arachidonic acid (AA) and [^3^H] oleic acid (OA) incorporation into lipids

To evaluate the consequences of incubation with iron and copper on the label distribution among dopaminergic neuron lipid classes, cells were pre-labeled with [^3^H] arachidonic acid (AA, 20:4) or [^3^H] oleic acid (OA, 18:1). The labeled fatty acid (0.5 μCi per well) was mixed with unlabeled AA (1.5 μM) in the presence of lipid-free BSA (4 mol AA/mol BSA). Cells were pre-incubated for 5 h at 37°C with [^3^H] AA or [^3^H] OA supplemented medium to allow the labeled fatty acid incorporation. After this incubation, the medium was removed and replaced with serum free medium and cells were treated with 1 mM Fe^2+^, 50 μM Cu^2+^, the combination of these metals or the vehicle for 24 h.

After treatments, cells were washed 3 times with PBS, scraped off and subjected to lipid extraction following the method of Bligh and Dyer [24]. Lipid extracts were washed 2 times with Bligh and Dyer upper phase, dried under N_2_, resuspended in chloroform/methanol (2:1, vol/vol) and spotted on silica gel plates. For the resolution of neutral lipids, silica gel G plates were used and the mobile phase consisted in hexane: eter: acetic acid (50: 50: 2.6). In order to resolve polar lipids, lipid extracts were spotted on silica gel H plates and phospholipids were separated by two dimensional thin layer chromatography exactly as described before [23]. Lipid spots were visualized by exposing the plates to iodine vapors. The regions corresponding to monoacylglycerols (MAG), diacylglycerols (DAG), free fatty acids (FFA) and triacylglycerols (TAG) were scraped off the silica gel G plates. The regions corresponding to phosphatidylcholine (PC), phosphatidylethanolamine (PE), phosphatidylserine (PS), phospatidylinositol (PI) and phosphatidic acid (PA) were scraped off the silica gel H plates. All spots were transferred to vials where silica was deactivated by the addition of water. Five milliliters of 0.4% (wt/vol) Omnifluor in toluene/Arkopal N-100 (4:1, vol/vol) were subsequently added. Radioactivity in lipid spots from the blanks (typically less than 100 dpm) was subtracted from those of experimental samples.

### Lipid droplets staining

After treatments, cells were washed 3 times with M1 buffer (150 mM NaCl, 1 mM CaCl_2_, 1 mM MgCl_2_, and 5 mM KCl in 20 mM HEPES buffer, pH 7.4) and stained for 15 min with Nile Red (Molecular Probes), 1.5 μg/ml in M1 buffer. After rinsing three times with M1 buffer, cells were observed with a Nikon Eclipse E-600 microscope (Nikon, Melville, NY, USA), using a K2E Apogee CCD camera driven by CCDOPS software (Santa Barbara Instrument Group, Santa Barbara, CA, USA) to visualize LD.

### Determination of *Plin-2* mRNA levels

Total RNA was isolated from dopaminergic neurons after the corresponding treatments using TRIZOL Reagent (Invitrogen) according to the manufacturer's protocol. RNA was resuspended in nuclease-free water and its concentration was assessed from A260:A280 absorbance ratio in a PicoDrop Spectrophotometer. Samples were stored at −80°C until use. Aliquots containing 2 μg total RNA were used to synthesize cDNA in reactions containing 1 μg Random Primer hexamers (Biodynamics), 1X M-MLV Reverse Transcriptase Reaction Buffer, 0.5 mM of each dNTP, 25 UI RNase inhibitor (Promega) and 200 UI M-MLV Reverse Transcriptase (Promega). Reactants were taken to a final volume of 25 μl with RNase-free water.

The cDNA resulting from RT was amplified by real-time quantitative PCR (qPCR). Gene expression levels were determined using Rotor-Gene 6000 (Corbett Research, Australia). RT-qPCR was performed in a final volume of 10 μl using Mezcla Real for Real-Time PCR (Biodynamics) and 0.2 μM of each primer. Gene-specific primer*s* sequences designed for RT-qPCR were to *Plin-2*, forward: gtgacctcagtggctgtgac and reverse: tattggcgaccgcaatct and to *Tbp*, forward: tgggattgtaccacagctcca and reverse: ctcatgatgactgcagcaaacc and both primer pairs were purchased from Life Technologies. PCR conditions were as follows: 40 cycles of denaturation at 94°C for 20 s, annealing and extension at 58°C for 20 s and a final extension step at 72°C for 30 s. Ct values of *Plin-2* mRNA obtained from 3 different experiments were normalized according to the 2^-ΔΔCt^ method, using TATAA-box binding protein (*Tbp*) as a reference gene. At the end of the amplification phase, a melting curve analysis was carried out on the product formed and agarose gel electrophoresis was carried out to confirm product size. The relative level of *Plin-2* is expressed as the relative change in gene expression.

### Triacylglycerol and cholesterol measurement

After separation by TLC, the spots corresponding to TAG and cholesterol were scrapped off the silica and eluted. Total TAG and cholesterol content was measured in aliquots of the lipid extracts corresponding to 10 μg lipid P, using commercial kits (TG color GPO/PAP AA and Colestat enzimático) from Wiener laboratory. Aliquots were dried under N_2_ gas and resuspended in 100 μl of isopropyl alcohol and the manufacturer’s instructions were subsequently followed.

### Statistical analysis

Data represent the mean value ± SD of at least three independent experiments, each experiment was performed in triplicate. Statistical significance was determined by either Student’s t-test or two-way ANOVA followed by a Tukey’s test. p-values lower than 0.05 were considered statistically significant. For cytochemical studies, 10 fields per sample were analyzed in each case. The Western Blots shown are a representative image of samples from three independent experiments.

## Results

### Effect of Fe^2+^ overload and Cu^2+^ supplementation on dopaminergic neurons

**Fig 1:**
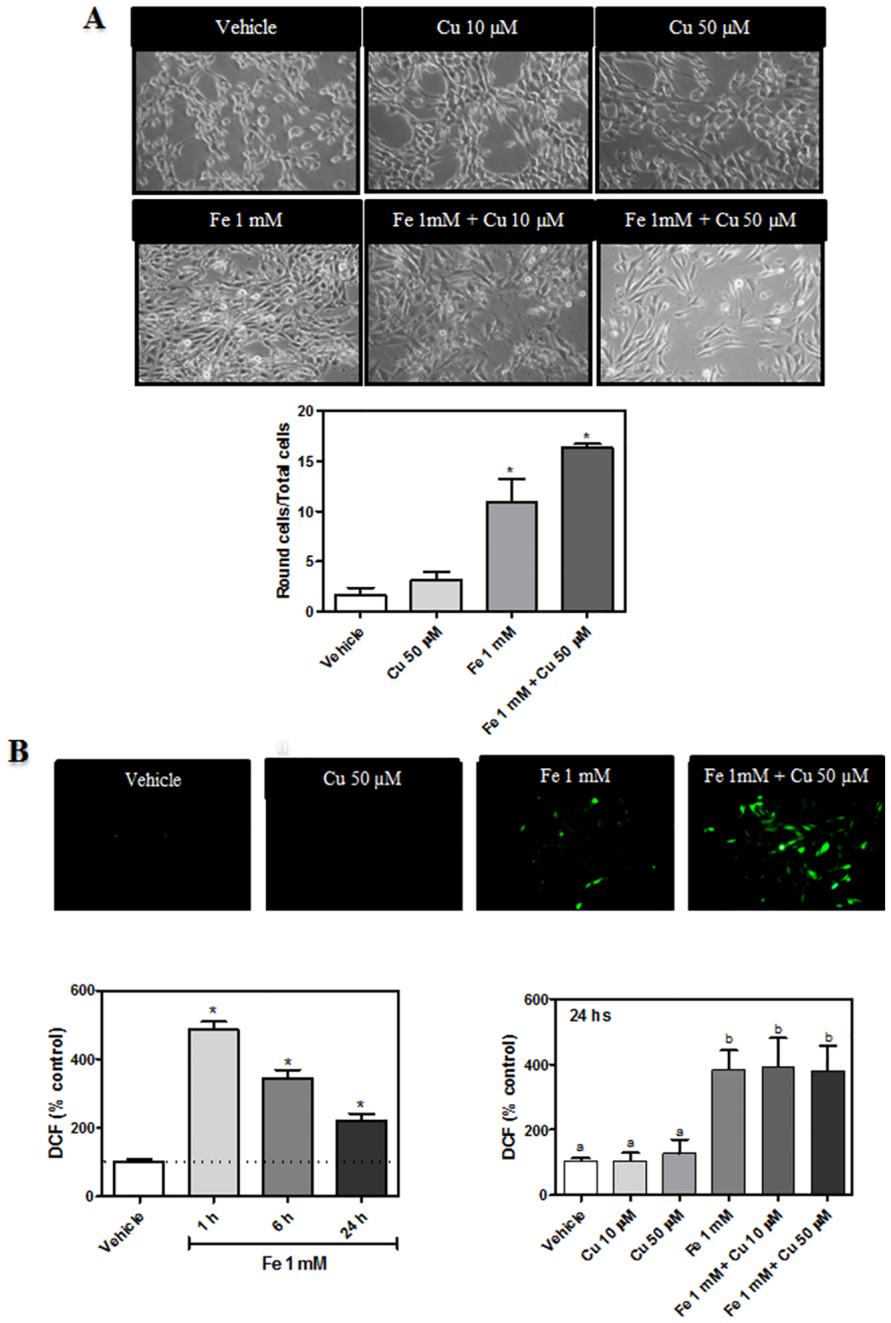
Effect of Fe^2+^ overload and Cu^2+^ supplementation on dopaminergic neurons. (A) Photomicrographs of cells after the exposure to Cu^2+^ (10, 50 μM), Fe^2+^ overload (1 mM), the combination of both metals or its vehicle for 24 h. Representative images from three different experiments are shown. (B) Determination of cellular oxidant levels (ROS). Cells were treated as described in A and subsequently incubated in the presence of 10 μM DCDCDHF. ROS generation by Fe^2+^ overload (1 mM) was also evaluated as a time function (0–24h). Cellular oxidant levels were imaged with an epifluorescence microscope and quantified by spectrofluorimetry. Results are expressed as a percentage of the control and represent mean ± SD (n = 3)

Our laboratory has previously characterized the effects of Fe-induced oxidative stress on cerebral cortex synaptic endings, retina and hippocampal neurons [21,22,25]. We have also described the activation of several signaling pathways related to lipid messengers during metal induced oxidative injury in the above-mentioned experimental models [19–21,26,27]. In the present work, we have characterized the role of PL and TAG acylation and remodeling during metal-induced toxicity in dopaminergic neurons. To this end, rat mesencephalic dopaminergic neurons, N27 cells, were exposed to Cu^2+^ (10, 50 μM), Fe^2+^ overload (1 mM) and the combination of both metals for 24 h. Control conditions were also assessed, replacing metals by an equal volume of vehicle (ultrapure water). To determine the extent of metal-induced effects on dopaminergic neurons, cell morphology, generation of reactive oxygen species (ROS), cell viability and lipid peroxidation levels were analyzed. Exposure to Cu^2+^ ions (10 and 50 μM) did not significantly alter cell morphology. However, Fe^2+^ (1 mM) caused typical morphological alterations of oxidant cellular injury, such as cytoplasmic retraction, cell body rounding and decrease in the number of cells and neuronal projections. These changes in cellular morphology were not affected when cells were incubated with the combinations Fe-Cu assayed (Fig 1A).

**Fig 2:**
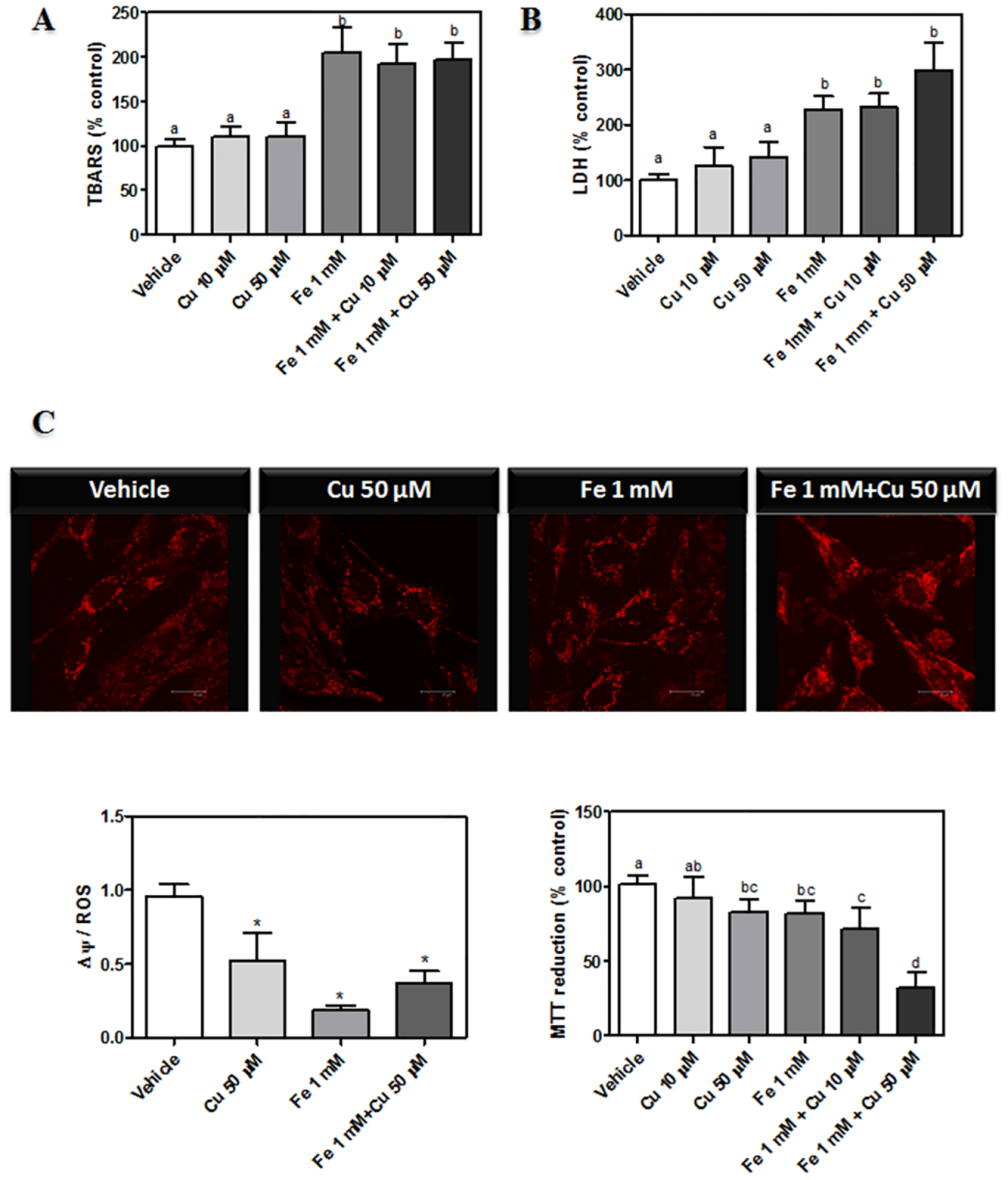
Effect of Fe^2+^ overload and Cu^2+^ supplementation on dopaminergic neurons. (A) TBARS assay. Cells were treated as described in A and lipid peroxidation was studied according to “Materials and Methods”. Results are expressed as a percentage of the control and represent mean ± SD (n = 8). (B) LDH leakage assay was assessed in cells after their exposure to the same conditions described in A. Results are expressed as a percentage of the control and represent mean ± SD (n = 10). (C) Mitochondrial characterization. Mitochondrial membrane potential (Δᴪ) was measured using the probe Mitotracker Red CMX-ROS and mitochondrial function was evaluated by MTT reduction assay. Cells were treated as described in A, mitochondria stained with Mitotracker were visualized by confocal microscopy and MTT reduction was assessed as specified in “Materials and Methods”. Δψ/ROS ratio was calculated for all the experimental conditions. Results are expressed as a percentage of the control and represent mean ± SD (n = 10). Values with different letters (a, b, c, d) are significantly different (p<0.05), one-way ANOVA and Tukey’s *post hoc* test.

A highly significant increase (300% higher than the controls) in cellular ROS (measured by using the probe DCDCDHF) was observed after the treatment with 1mM Fe^2+^ or with Fe-Cu combinations (1 mM-10 μM; 1 mM-50 μM). However, the presence of Cu^2+^ alone did not increase ROS generation (Fig 1B). Neurons incubated in the presence of iron showed a sustained increase in ROS levels ranging from 1 to 24 h of incubation. Lipid peroxidation levels, which were analyzed by TBARS assay, were significantly increased by the presence of Fe (1mM) and with the co-incubation of both metals (approximately 100%, higher than the control; Fig 2A). Co-incubation with Cu and Fe showed no increase of TBARS levels with respect to the Fe condition. As shown in Fig 2B, experimental conditions with increased TBARS formation showed an augmentation of cell membrane permeability measured by LDH leakage. Mitochondrial membrane potential (ΔΨ_m_) was also measured in the above mentioned experimental conditions, using the probe Mitotracker CMX-ROS (Fig 2C). In the presence of iron, ΔΨ_m_/ROS ratio strongly decreased, thus demonstrating mitochondrial depolarization. This fact agree with a decreased mitochondrial function, measured as MTT reduction (Fig 2C). TBARS and ROS generation were not increased in the presence of lower Fe^2+^ concentrations than 1 mM (data not shown). The fact that Fe^2+^ concentrations ranging from 10 to 200 μM produced no neuronal damage and that earlier times of incubation (1–6h) with 1 mM Fe^2+^ did not produce neuronal damage were the reasons why we exposed dopaminergic neurons to 1 mM Fe^2+^ for 24 hs. In line with this, similar concentrations were used by the study of α-syn toxicity in a cell model of PD [28].

Cell viability, assessed by MTT reduction assay, was diminished at 50 μM Cu, 1 mM Fe and the combinations of both metals assayed (Fig 2C).

### Phospholipid and triacylglycerol acylation in pre-labeled dopaminergic neurons exposed to metal-induced injury

**Fig 3:**
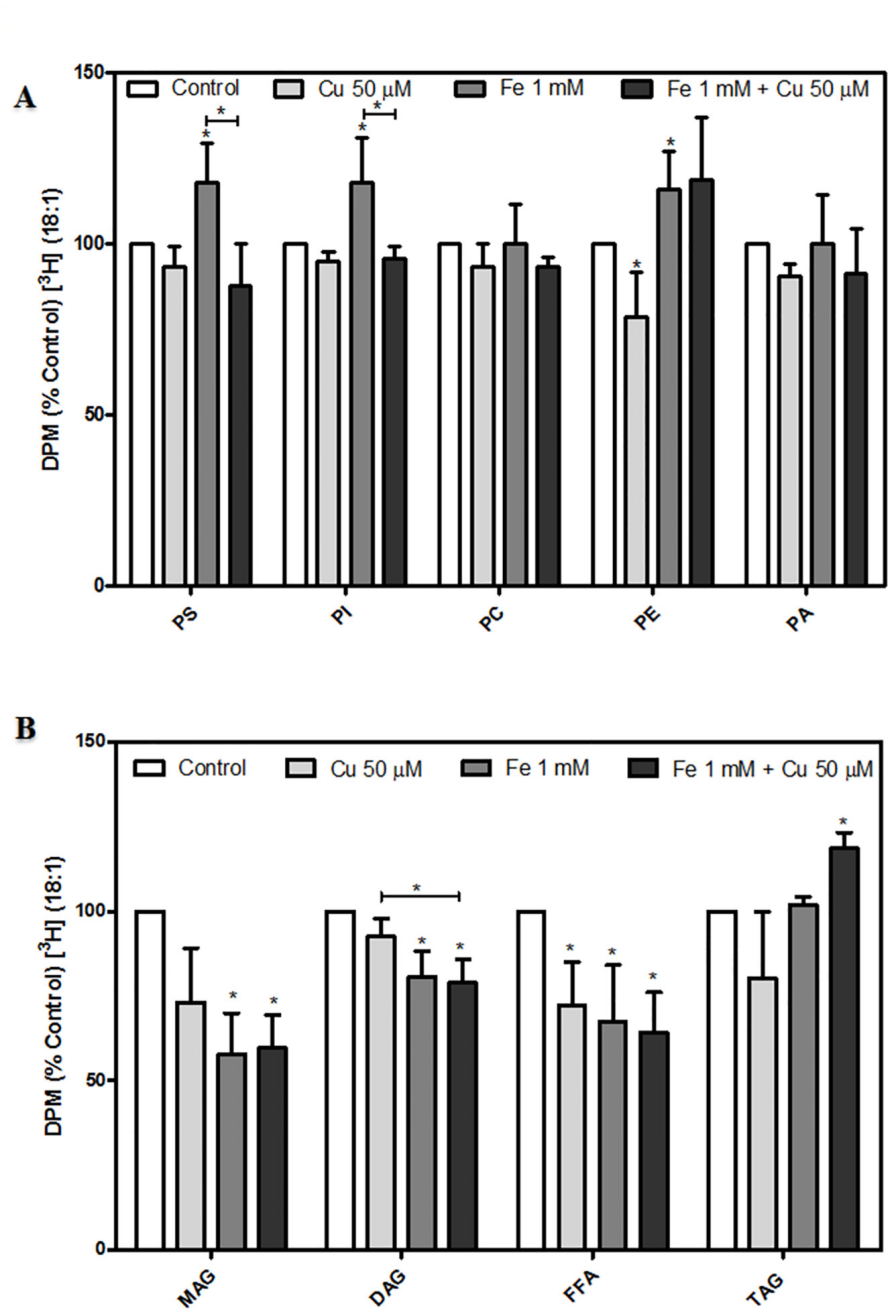
Effects of Fe^2+^ overload and Cu^2+^ supplementation on the distribution (%) of [^3^H] oleic acid (OA) among dopaminergic neuron lipids. [^3^H] oleic acid (OA, 18:1) incorporation into (A) Phospholipids (PS, PI, PC, PE and PA) and (B) Neutral lipids (MAG, DAG, FFA and TAG) were analyzed after cells were pre-labeled with [^3^H] (OA) for 5 h at 37°C and subsequently treated with 50 μM Cu^2+^, 1 mM Fe^2+^, the combination of these metals or the vehicle for 24 h as described in “Materials and Methods”. Results are expressed as a percentage of the control of each lipid and represent mean ± SD (n = 3). *p < 0.05 for each condition with respect to the control; one-way ANOVA and Tukey’s *post hoc* test.

Pre-labeled dopaminergic neurons with [^3^H] oleic acid (OA, 18:1) or [^3^H] arachidonic acid (AA, 20:4) showed a distinctive profile of FA incorporation into PL and TAG. During Fe-induced injury, OA incorporation was increased in PS, PI and PE (Fig 3A). OA incorporation into TAG from Fe-exposed neurons showed no changes with respect to control conditions. whereas its incorporation into MAG and DAG decreased significantly (Fig 3B).

**Fig 4:**
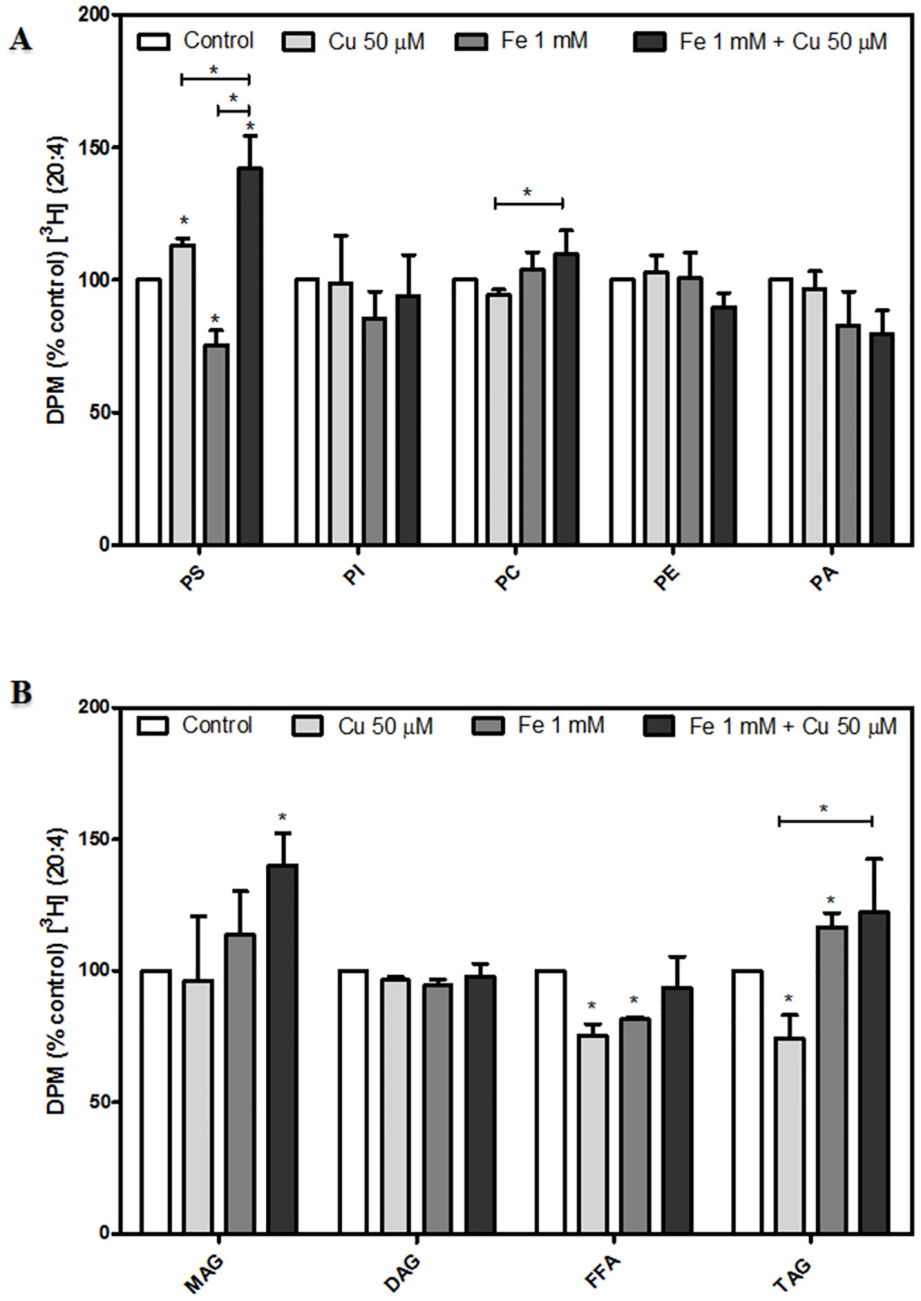
Effects of Fe^2+^ overload and Cu^2+^ supplementation on the distribution (%) of [^3^H] arachidonic acid (AA) among dopaminergic neuron lipids. [^3^H] arachidonic acid (AA, 20:4) incorporation in (A) Phospholipids (PS, PI, PC, PE and PA) and (B) Neutral lipids (MAG, DAG, FFA and TAG) was analyzed after cells were prelabeled with [^3^H] AA for 5 h at 37°C and treated with 50 μM Cu^2+^, 1 mM Fe^2+^, the combination of these metals or the vehicle for 24 hours as described in “Materials and Methods”. Results are expressed as a percentage of the control of each lipid and represent mean ± SD (n = 3). *p < 0.05 for each condition with respect to the control; one-way ANOVA and Tukey’s *post hoc* test.

Iron-exposed neurons showed decreased levels of AA uptake into PS with respect to control (Fig 4A). In contrast, AA incorporation into TAG was increased whereas decreased free AA levels were observed under the same experimental conditions (Fig 4B). Cu-incubations did not affect OA incorporation but decreased AA incorporation into TAG (Figs 3B and 4B). However, Cu-incubated neurons showed decreased OA incorporation into PE and increased AA uptake into PS (Figs 3A and 4A).

### TAG levels in dopaminergic neurons exposed to metal-induced injury

**Fig 5:**
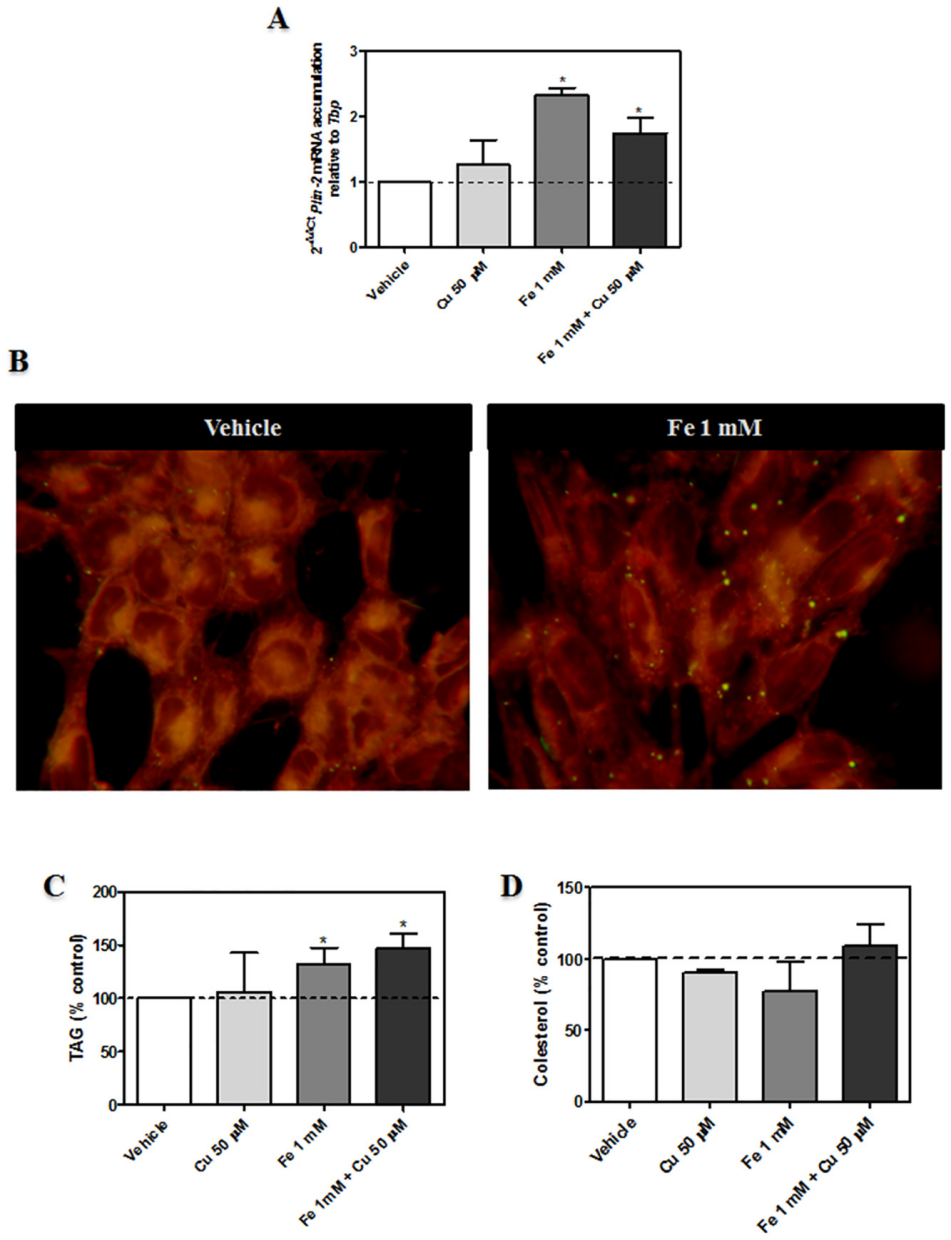
Effect of metal-induced injury on TAG content and formation of LD. (A) RT-qPCR analysis for *Plin-2* transcripts in N27 cells after incubation for 5 h with 1 mM Fe^2+^, 50 μM Cu^2+^, the combination of these metals or the vehicle. Data was normalized to *Tbp* as internal reference gen using the 2^-ΔΔCt^ method (n = 3). (B) Fluorescence images of dopaminergic neurons exposed to 1 mM Fe^2+^, or the vehicle for 24 hours, stained with Nile Red to detect LD. Representative images from three different experiments are shown. (C) TAG content in N27 incubated in the presence of 1 mM Fe^2+^, 50 μM Cu^2+^, the combination of these metals or the vehicle for 24 hours. Results are expressed as a percentage of the control and represent mean ± SD (n = 3). (D) Cholesterol content in N27 incubated in the presence of 1 mM Fe^2+^, 50 μM Cu^2+^, the combination of these metals or the vehicle for 24 hours. Results are expressed as a percentage of the control and represent mean ± SD (n = 3). *p < 0.05 for each condition with respect to the control; one-way ANOVA and Tukey’s *post hoc* test.

**Fig 6:**
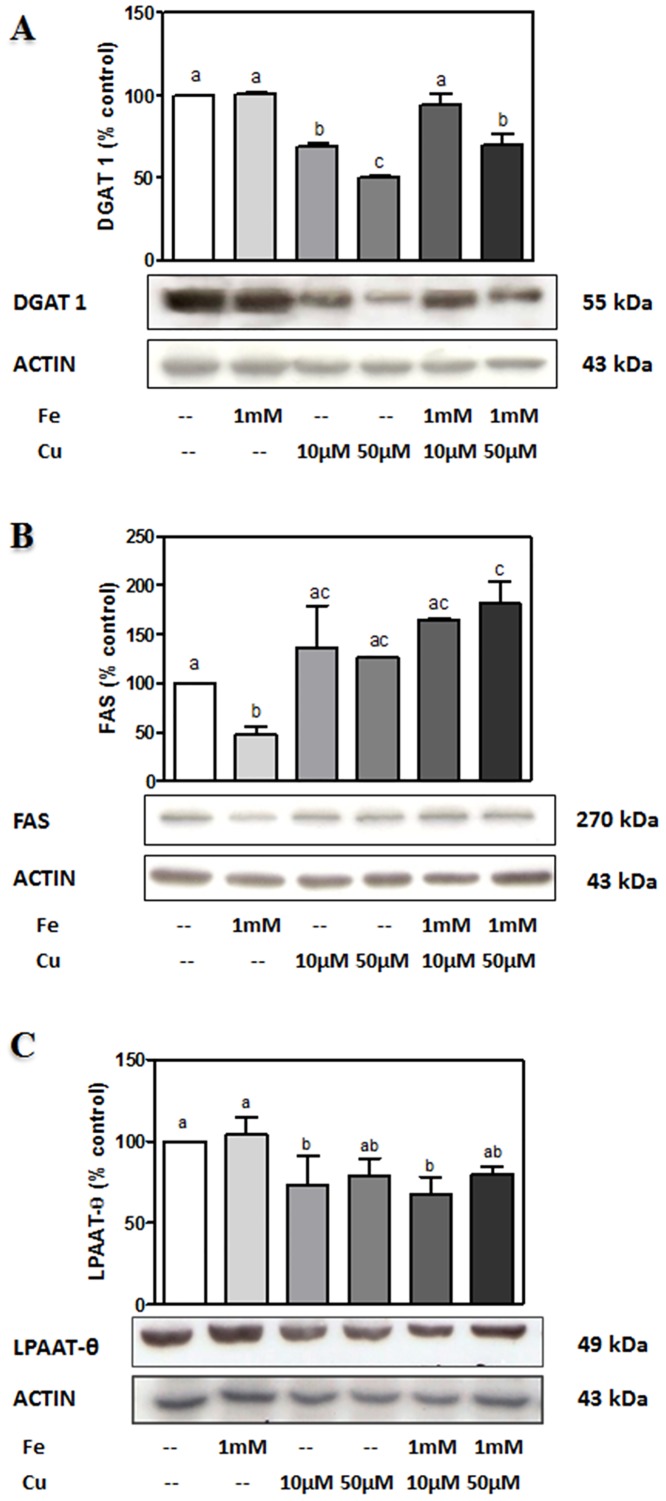
Western blot analyses of the enzymes involved in lipid accumulation. Cells were exposed to Cu^2+^ (10, 50 μM), Fe^2+^ overload (1 mM), the combination of both metals or its vehicle for 24 h. Cell lysates were prepared for Western blot as described in “Material and Methods” and (A) DGAT 1 expression levels, (B) FAS expression levels and (C) LPAAT-θ expression levels were assessed. Western blot in each case is representative of three different experiments. Bands of proteins were quantified using scanning densitometry. Data in the graphs above each blot represent the ratio between cellular protein expression and Actin, expressed as a percentage of the corresponding control condition (mean ± SD of three different experiments). *p < 0.05 for each condition with respect to the control; one-way ANOVA and Tukey’s *post hoc* test.

The increase observed in TAG-containing AA in Fe-exposed neurons was accompanied by an increase in mRNA levels of the LD protein Perilipin-2 (PLIN-2), determined by RT-qPCR (Fig 5A). In the presence of Fe, Nile red positive cytoplasmic bodies, were also observed (Fig 5B). This was coincident with increased TAG levels being 40% higher in Fe-exposed neurons with respect to controls (Fig 5C). Cu did not produce any increase neither in LD appearance nor in TAG levels. Cholesterol levels were not changed in any of the experimental conditions tested (Fig 5D). For further characterizing the mechanisms involved in AA accumulation in TAG during Fe-induced injury, the expression of DGAT 1, lysophosphatidic acid acyltransferase θ (LPAAT θ) and fatty acid synthase (FAS) proteins were analyzed by Western Blot. Intriguingly, DGAT 1 levels were found to undergo no changes in Fe-exposed neurons whereas Cu-exposed neurons were observed to decrease the levels of this enzyme (Fig 6A). FAS protein levels were diminished in Fe-exposed neurons and augmented in Cu-exposed neurons, with respect to controls and LPAAT-θ levels underwent no changes in any of the experimental conditions analyzed (Fig 6B and 6C).

### Role of lipid acylation/deacylation mechanisms in dopaminergic neurons exposed to metal-induced injury

**Fig 7:**
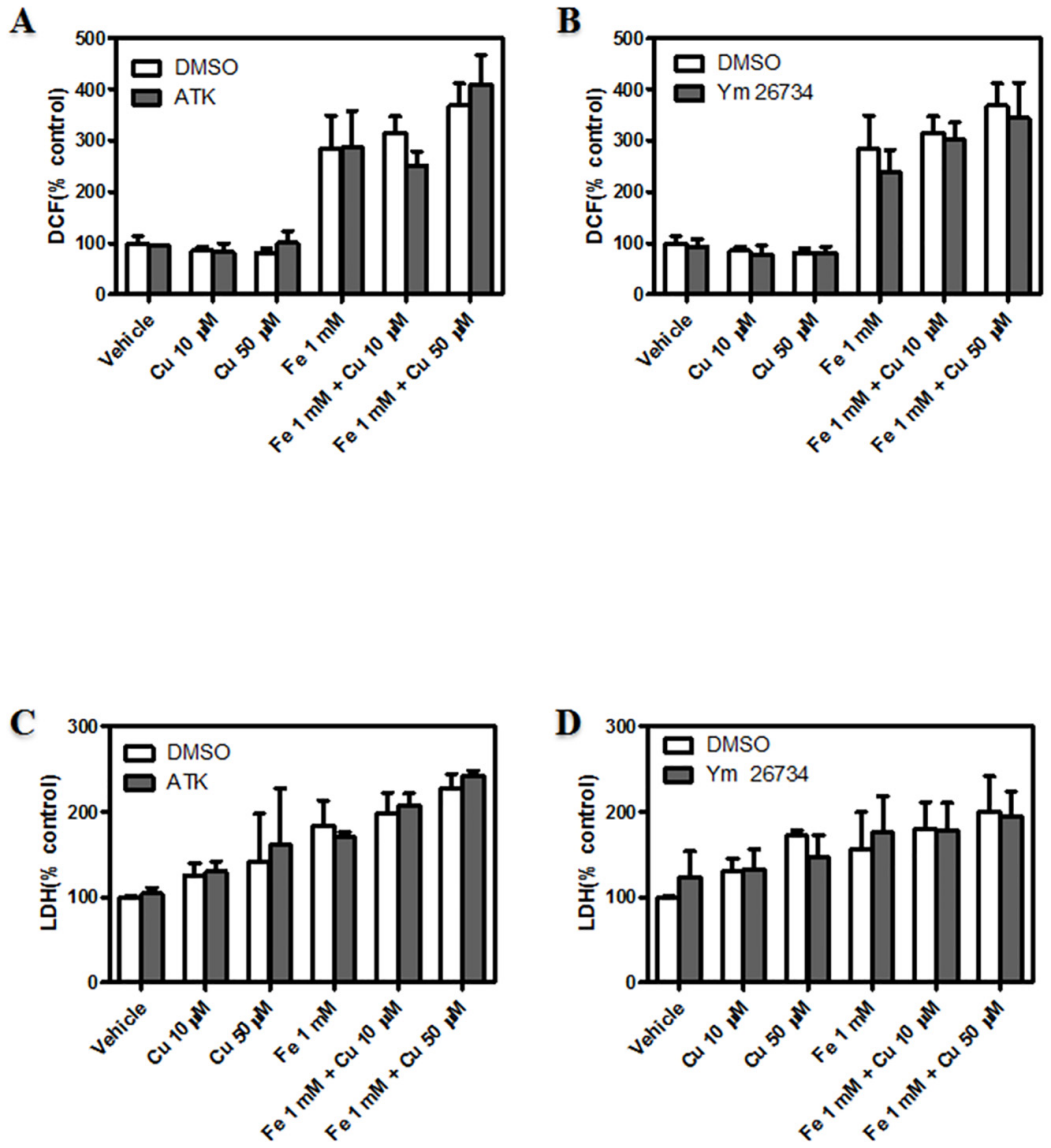
Role of lipid deacylation mechanisms mediated by sPLA_2_ and cPLA_2_ in dopaminergic neurons exposed to metal-induced injury. (A) Determination of cellular oxidant levels. Cells were treated with Cu^2+^ (10, 50 μM), Fe^2+^ overload (1 mM), the combination of both metals or its vehicle for 24 hs in the presence or absence of cPLA_2_ inhibitor (ATK 50 μM), and subsequently incubated in the presence of 10 μM DCDCDHF. Cellular oxidant levels were quantified by spectrofluorimetry. Results are expressed as a percentage of the control and represent mean ± SD (n = 3). (B) Determination of cellular oxidant levels. Cells were treated with Cu^2+^ (10, 50 μM), Fe^2+^ overload (1 mM), the combination of both metals or its vehicle for 24 hs in the presence or absence of sPLA_2_ inhibitor (Ym 26734 10 μM), and subsequently incubated in the presence of 10 μM DCDCDHF. Cellular oxidant levels were quantified by spectrofluorimetry. Results are expressed as a percentage of the control and represent mean ± SD (n = 3). (C) LDH leakage assay was assessed in cells after their exposure to Cu^2+^ (10, 50 μM), Fe^2+^ overload (1 mM), the combination of both metals or its vehicle for 24 h in the presence or absence of cPLA_2_ inhibitor (ATK 50 μM) Results are expressed as a percentage of the control and represent mean ± SD (n = 3). (D) LDH leakage assay was assessed in cells after their exposure to Cu^2+^ (10, 50 μM), Fe^2+^ overload (1 mM), the combination of both metals or its vehicle for 24 h in the presence or absence of sPLA_2_ inhibitor (Ym 26734 10 μM). Results are expressed as a percentage of the control and represent mean ± SD (n = 3). *Significantly different compared to the respective control group (p<0.05, one-way ANOVA test followed by Tukey *post hoc* test)

**Fig 8:**
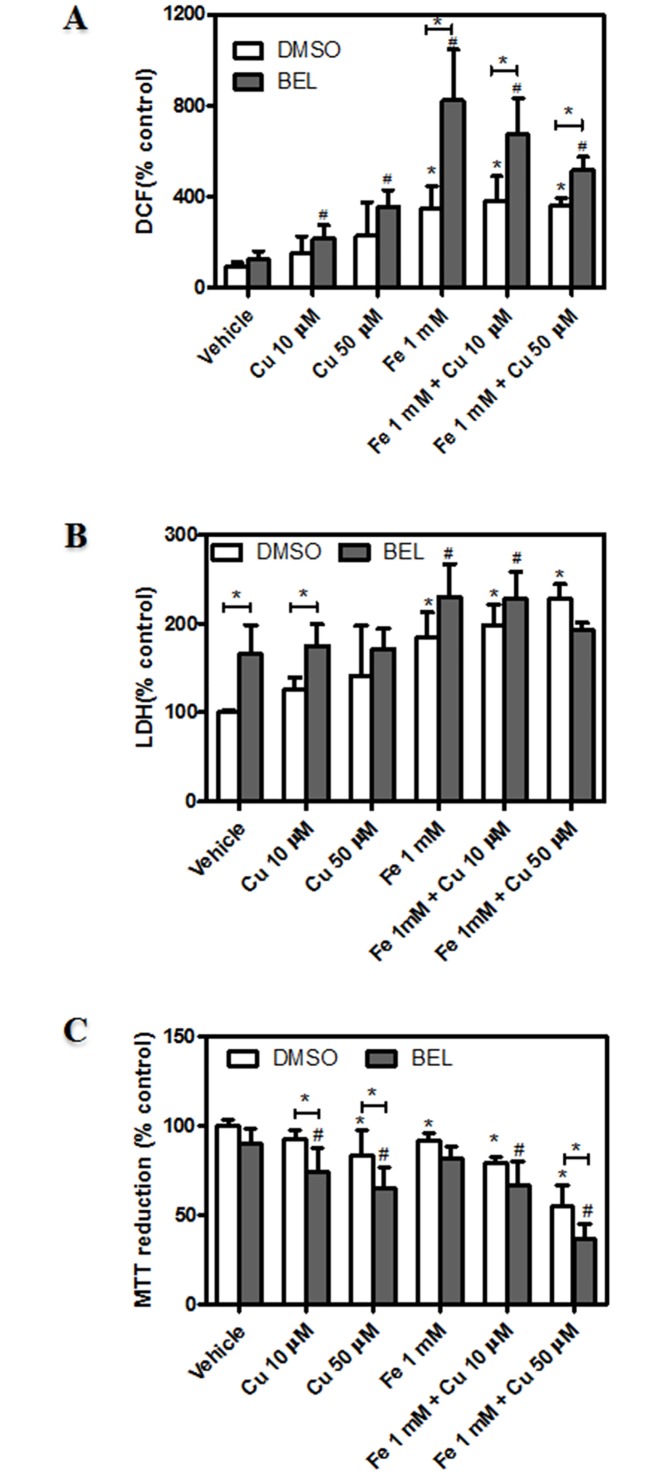
Role of lipid deacylation mechanisms mediated by iPLA_2_ in dopaminergic neurons exposed to metal-induced injury. (A) Determination of cellular oxidant levels. Cells were treated with Cu^2+^ (10, 50 μM), Fe^2+^ overload (1 mM), the combination of both metals or its vehicle for 24 hs in the presence or absence of iPLA_2_ inhibitor (10 μM BEL) and subsequently incubated in the presence of 10 μM DCDCDHF as described in “Materials and Methods”. Cellular oxidant levels were quantified by spectrofluorimetry. Results are expressed as a percentage of the control and represent mean ± SD (n = 3). (B) LDH leakage assay was assessed in cells treated as described in A. Results are expressed as a percentage of the control and represent mean ± SD (n = 3–4). (C) MTT reduction assay. Cells were treated as described in A and cell viability was assessed as described in “Materials and Methods”. Results are expressed as a percentage of the control and represent mean ± SD (n = 3). *Significantly different compared to the respective control group (p<0.05, one-way ANOVA test followed by Tukey *post hoc* test). #Significantly different compared to the respective control group (in the presence of iPLA_2_ inhibitor, 10 μM BEL)

Based on the results obtained in lipid acylation process during metal-overload, and in order to further investigate the far-reaching implication of lipid remodeling during metal-induced neuronal injury, we next investigated the involvement of deacylation and acylation processes in ROS generation and membrane permeability. To this end, PLA_2_ isoform-specific inhibitors and a general inhibitor of lipid acylation (CI-976) were added to the above-mentioned assays. Dopaminergic neurons were preincubated with 50 μM ATK (cPLA_2_ inhibitor), 10 μM YM 26734 (sPLA_2_ inhibitor) and 10 μM BEL (iPLA_2_ inhibitor) and subsequently exposed to metal incubations. cPLA_2_ and sPLA_2_ inhibition did not alter the increase in ROS generation observed in the presence of Fe, Cu and the combination of both (Fig 7A and 7B). Neither membrane permeability (Fig 7C and 7D) nor mitochondrial function (data not shown) were affected by the inhibition of cPLA_2_ and sPLA_2_. However, whereas iPLA_2_ inhibition was found to strongly increase the generation of ROS in the presence of Fe-induced injury (Fig 8A), membrane permeability and mitochondrial function were slightly affected by iPLA_2_ inhibition (Fig 8B and 8C).

**Fig 9:**
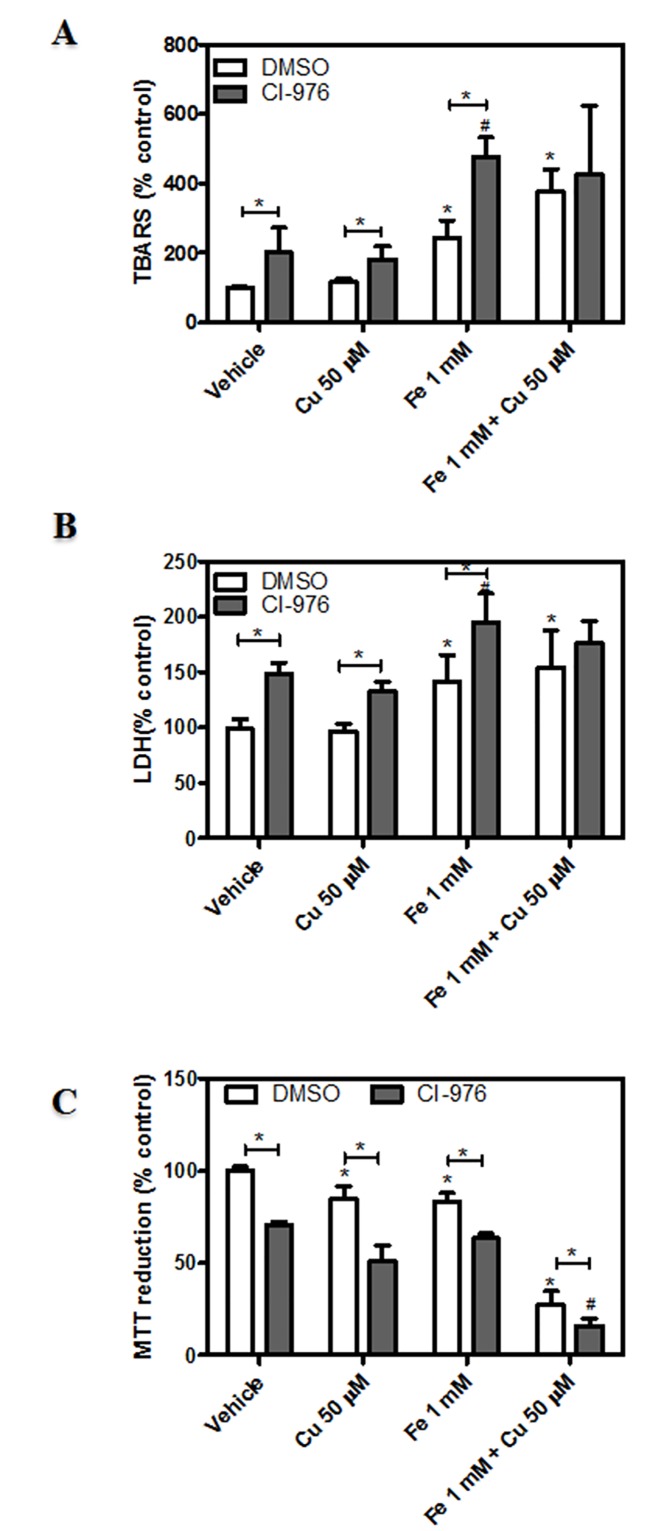
Role of lipid acylation mechanisms in dopaminergic neurons exposed to metal-induced injury. (A) Determination of cellular oxidant levels. Cells were treated with Cu^2+^ (50 μM), Fe^2+^ overload (1 mM), the combination of both metals or its vehicle for 24 hs in the presence or absence of the acylation inhibitor (7.5 μM CI-976) and subsequently incubated in the presence of 10 μM DCDCDHF as described in “Materials and Methods”. Cellular oxidant levels were quantified by spectrofluorimetry. Results are expressed as a percentage of the control and represent mean ± SD (n = 3). (B) LDH leakage assay was assessed in cells treated as described in A. Results are expressed as a percentage of the control and represent mean ± SD (n = 3–4). (C) MTT reduction assay. Cells were treated as described in A and cell viability was assessed as described in “Materials and Methods”. Results are expressed as a percentage of the control and represent mean ± SD (n = 3). *Significantly different compared to the respective control group (p<0.05, one-way ANOVA test followed by Tukey *post hoc* test)

The inhibition of acyltransferase activities did not alter ROS levels under any of the conditions assayed (data not shown). Nevertheless, lipid peroxide generation and membrane permeability were observed to be increased by metal-induced neuronal injury in the presence of CI-976 with respect to controls (Fig 9A and 9B). This was in accordance with a significant loss of mitochondrial function (Fig 9C).

## Discussion

The mechanisms that operate during dopaminergic neuron injury are a major concern for understanding the ethiopathogenesis of PD. Excessive iron content and the resulting oxidative stress in the *substancia nigra* of PD patients are considered triggering factors in this pathology [2,29,30]. A less clear issue is the state of Cu metabolism [1] although recent findings have demonstrated that α-syn pathology enhances vulnerability to cell death if Cu-dependent protective mechanisms are impaired [9]. In this respect, an unexplored field is how lipid acylation/deacylation cycles operate during dopaminergic neuron degeneration and death. Therefore, for piecing together the above-mentioned events, lipid acylation mechanisms and their role in oxidative dopaminergic injury triggered by Fe-overload were studied.

Fe-exposed N27 neurons were observed to display increased levels of lipid peroxides and ROS and also augmented membrane permeability. As a result, Fe-overloaded neurons exhibited slightly diminished mitochondrial viability. Our results as well as previous evidence centering on the role of Fe in dopaminergic degeneration support the viability of the experimental model used in the present study [10,31]. In line with this, advances in non-dopaminergic treatments for PD are focused in the use of Fe chelators as therapeutic strategy [32]. The condition of Cu supplementation induced no increase in oxidative stress markers although at the highest Cu concentration tested, a reduced mitochondrial function was observed. These findings suggest that Fe-overload triggers dopaminergic neuron injury by oxidative stress whereas Cu-mediated dopaminergic neurotoxicity does not involve oxidative injury, at least at the concentrations assayed in the present study.

Neuronal phospholipid acylation and deacylation mechanisms are crucial for the maintenance of axonal and synaptic integrity [33]. Phospholipid membrane deacylation is catalyzed by different isoforms of PLA_2_ that generate AA, docosahexaenoic acid and lysophospholipids. All these bioactive lipids have a variety of physiological effects and also behave as substrates for the synthesis of other potent lipid mediators, such as eicosanoids, and the lipid peroxides, 4-hydroxynonenal and malondialdehyde. Rapid clearance of peroxidized lipids is a necessary mechanism for preserving plasma membrane integrity during oxidative stress processes [34–36]. Our results show a different lipid acylation profile during metal-induced injury. The increase of OA acylation in PS, PI and PE and the decrease of AA incorporation into PS demonstrate that dopaminergic neurons respond to Fe-induced oxidative injury by switching PL fatty acid composition to a more saturated profile. The increased preference of OA as an acyl donor for PL acylation could be indicative of the fact that dopaminergic neurons decreased PUFA levels in their membranes in order to be less prone to peroxidation. Our results agree with previous findings according to which lipids containing arachidonate are more susceptible to oxidative degradation [37,38]. These findings are, in turn, in agreement with the membrane pacemaker hypothesis on ageing, which proposes that animals whose cellular membranes contain high amounts of PUFA have shorter life spans because of their susceptibility to oxidative stress [39]. In Cu-exposed membranes, PL acylation was opposite to the profile observed in Fe-exposed neurons as it increased the incorporation of AA into PS –whereas it decreased OA labeling in PE. The absence of lipid peroxides in Cu-exposed neurons justify the incorporation of AA into PS, one of the main reservoirs for this FA in the brain [40].

Differential roles for specific-PLA_2_ isoforms have been described in several neurodegeneration processes. It has, in fact, been shown that recessively-inherited deficiency in the catalytic domain of iPLA_2_ causes neuroaxonal dystrophy [41]. A role for cPLA_2_ has particularly been observed to be involved in the generation of ROS through AA release in cerebral ischemia events and iron-induced retinal degeneration [42]. In addition, PL fatty acid composition and PLA_2_ activity have been shown to be related with the progression of AD [43]. In our study, oxidative injury markers, such as ROS content, lipid peroxide levels and membrane permeability, showed to be increased by the inhibition of iPLA_2_ (BEL). Furthermore, whereas PLA_2_s was found to deacylate PL in sn-2 position, AT activities were found to be necessary to reacilate this position as well as to maintain normal phospholipid composition [13]. Previous research also demonstrated that the loss of lysophosphatidylcholine acyltranferase that renders increased levels of lysophosphatodylcholine generates membrane disruption and Ca^2+^ influx [44]. Loss of lysophosphatidylcholine acyltransferase 1 was found to induce photoreceptor degeneration in rd11 mice [45]. The inhibition of PL acylation processes by CI-976 was observed to yield the same effect as that of BEL on Fe-exposed neurons. These observations highlight the crucial role of lipid acylation/deacylation processes in the response of dopaminergic neurons to oxidative injury.

The most striking finding of the present study seems to be the esterification of AA into TAG in Fe-overloaded neurons. The increase in TAG levels and the appearance of LD suggest that dopaminergic neurons respond to oxidative injury by the storage of peroxidable fatty acids in the TAG pool, thus avoiding the permanence of PUFA in PL fraction. The decreased expression of FAS also strengthens the hypothesis that under oxidative stress conditions dopaminergic neurons avoid the generation of new synthetized FA and exacerbate the mechanisms for preserving their FA from peroxidation. This strategy seemed to be successful on account of the fact that Fe-overloaded neurons were found to avoid a significant death. Regarding PD experimental models, rotenone, a mitochondrial toxicant, also triggers TAG accumulation in C2C12 cells [46].

**Fig 10:**
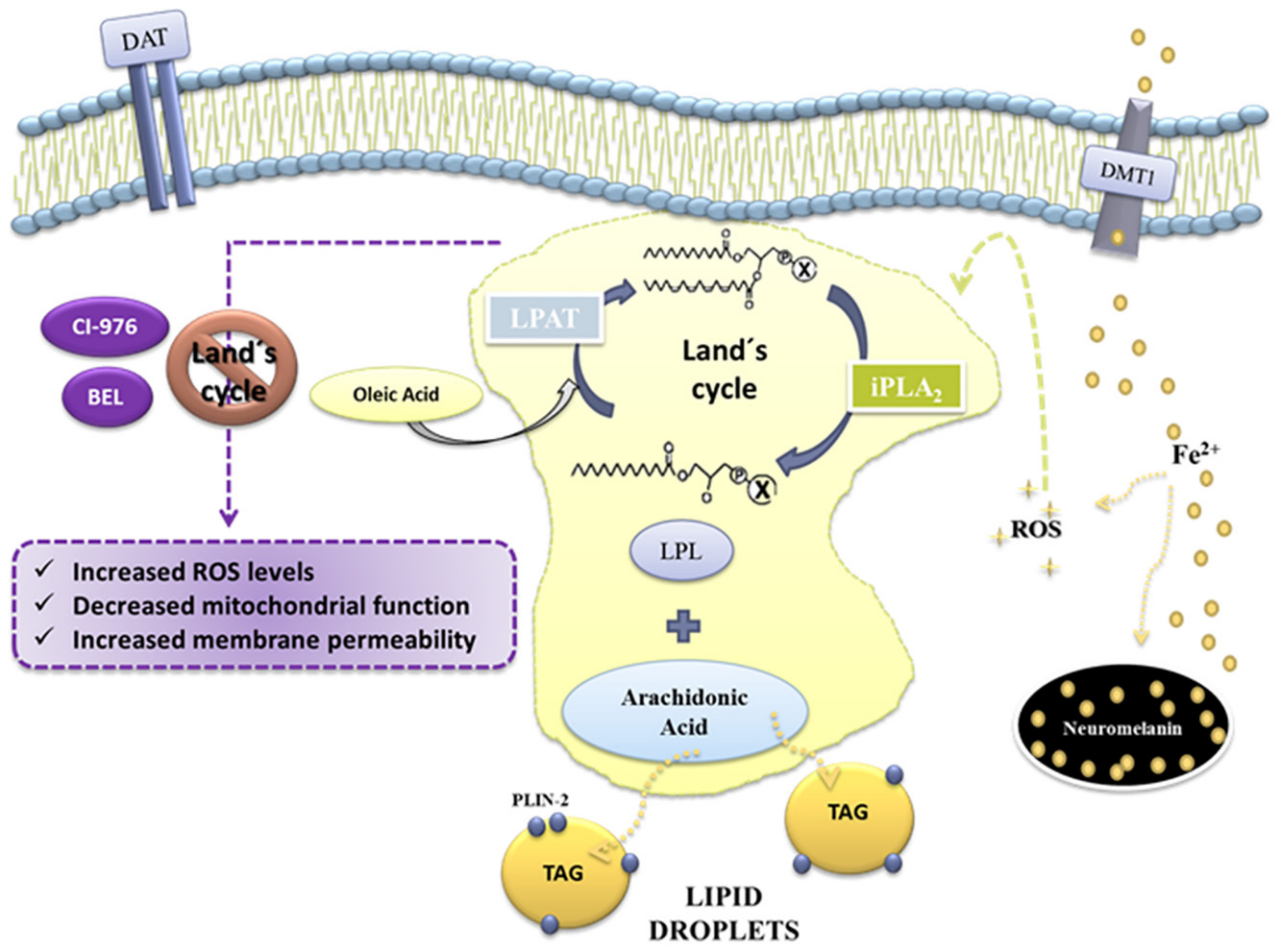
Lipid acylation mechanisms in Fe-induced dopaminergic neurons injury. Fe-induced oxidative stress triggers the accumulation of AA into TAG and the increase of OA-containing PL. The inhibition of lipid acylation/deacylation mechanisms exacerbates Fe-induced dopaminergic damage.

Taken together, findings from the present study provide evidence about the participation of lipid acylation mechanisms against Fe-induced oxidative stress in N27 cells and postulate that dopaminergic neurons strategically preserve AA pool in TAG in response to oxidative injury (Fig 10). Further studies in this direction will provide new knowledge on PD pathogenesis which could pave the way towards new preventive or therapeutic strategies.

## References

[1] Barnham KJ, Bush AI (2008) Metals in Alzheimer's and Parkinson's diseases. Curr Opin Chem Biol 12: 222–228. S1367-5931(08)00041-0 [pii]; 10.1016/j.cbpa.2008.02.019 18342639

[2] Dias V, Junn E, Mouradian MM (2013) The role of oxidative stress in Parkinson's disease. J Parkinsons Dis 3: 461–491. B5502M7312389W87 [pii]; 10.3233/JPD-130230 24252804 PMC4135313

[3] Kell DB (2010) Towards a unifying, systems biology understanding of large-scale cellular death and destruction caused by poorly liganded iron: Parkinson's, Huntington's, Alzheimer's, prions, bactericides, chemical toxicology and others as examples. Arch Toxicol 84: 825–889. 10.1007/s00204-010-0577-x 20967426 PMC2988997

[4] Raha AA, Vaishnav RA, Friedland RP, Bomford A, Raha-Chowdhury R (2013) The systemic iron-regulatory proteins hepcidin and ferroportin are reduced in the brain in Alzheimer's disease. Acta Neuropathol Commun 1: 55 2051-5960-1-55 [pii]; 10.1186/2051-5960-1-55 24252754 PMC3893417

[5] Devos D, Moreau C, Dujardin K, Cabantchik I, Defebvre L, Bordet R (2013) New pharmacological options for treating advanced Parkinson's disease. Clin Ther 35: 1640–1652. S0149-2918(13)00891-6 [pii]; 10.1016/j.clinthera.2013.08.011 24011636

[6] Gal S, Fridkin M, Amit T, Zheng H, Youdim MB (2006) M30, a novel multifunctional neuroprotective drug with potent iron chelating and brain selective monoamine oxidase-ab inhibitory activity for Parkinson's disease. J Neural Transm Suppl 447–456. 17017567 10.1007/978-3-211-45295-0_68

[7] Zhu Y, Hoell P, Ahlemeyer B, Sure U, Bertalanffy H, Krieglstein J (2007) Implication of PTEN in production of reactive oxygen species and neuronal death in in vitro models of stroke and Parkinson's disease. Neurochem Int 50: 507–516. S0197-0186(06)00326-3 [pii]; 10.1016/j.neuint.2006.10.010 17169462

[8] Howitt J, Gysbers AM, Ayton S, Carew-Jones F, Putz U, Finkelstein DI, Halliday GM, Tan SS (2014) Increased Ndfip1 in the substantia nigra of parkinsonian brains is associated with elevated iron levels. PLoS One 9: e87119 10.1371/journal.pone.0087119;PONE-D-13-33740 [pii]. 24475238 PMC3901732

[9] Davies KM, Bohic S, Carmona A, Ortega R, Cottam V, Hare DJ, et al(2014) Copper pathology in vulnerable brain regions in Parkinson's disease. Neurobiol Aging 35: 858–866. S0197-4580(13)00436-3 [pii]; 10.1016/j.neurobiolaging.2013.09.034 24176624

[10] Double KL (2012) Neuronal vulnerability in Parkinson's disease. Parkinsonism Relat Disord 18 Suppl 1: S52–S54. S1353-8020(11)70018-9 [pii]; 10.1016/S1353-8020(11)70018-9 22166454

[11] Farooqui AA (2009) Lipid mediators in the neural cell nucleus: their metabolism, signaling, and association with neurological disorders. Neuroscientist 15: 392–407. 15/4/392 [pii]; 10.1177/1073858409337035 19666894

[12] Farooqui AA, Horrocks LA (2006) Phospholipase A2-Generated Lipid Mediators in the Brain: The Good, the Bad, and the Ugly. The Neuroscientist 12: 245–260. 16684969 10.1177/1073858405285923

[13] Perez-Chacon G, Astudillo AM, Balgoma D, Balboa MA, Balsinde J (2009) Control of free arachidonic acid levels by phospholipases A2 and lysophospholipid acyltransferases. Biochim Biophys Acta 1791: 1103–1113. S1388-1981(09)00193-0 [pii]; 10.1016/j.bbalip.2009.08.007 19715771

[14] Shindou H, Shimizu T (2009) Acyl-CoA:lysophospholipid acyltransferases. J Biol Chem 284: 1–5. R800046200 [pii]; 10.1074/jbc.R800046200 18718904

[15] Wilfling F, Haas JT, Walther TC, Farese RV Jr. (2014) Lipid droplet biogenesis. Curr Opin Cell Biol 29: 39–45. S0955-0674(14)00042-8 [pii]; 10.1016/j.ceb.2014.03.008 24736091 PMC4526149

[16] Gui YX, Xu ZP, Wen L, Liu HM, Zhao JJ, Hu XY (2013) Four novel rare mutations of PLA2G6 in Chinese population with Parkinson's disease. Parkinsonism Relat Disord 19: 21–26. S1353-8020(12)00307-0 [pii]; 10.1016/j.parkreldis.2012.07.016 23182313

[17] Lee YJ, Wang S, Slone SR, Yacoubian TA, Witt SN (2011) Defects in very long chain fatty acid synthesis enhance alpha-synuclein toxicity in a yeast model of Parkinson's disease. PLoS One 6: e15946 10.1371/journal.pone.0015946 21264320 PMC3019226

[18] Ruiperez V, Darios F, Davletov B (2010) Alpha-synuclein, lipids and Parkinson's disease. Prog Lipid Res 49: 420–428. S0163-7827(10)00029-9 [pii]; 10.1016/j.plipres.2010.05.004 20580911

[19] Uranga RM, Giusto NM, Salvador GA (2009) Iron-induced oxidative injury differentially regulates PI3K/Akt/GSK3beta pathway in synaptic endings from adult and aged rats. Toxicol Sci 111: 331–344. kfp152 [pii]; 10.1093/toxsci/kfp152 19608791

[20] Rodriguez DG, Sanchez CS, Giusto NM, Salvador GA (2013) Specific roles for Group V secretory PLA(2) in retinal iron-induced oxidative stress. Implications for age-related macular degeneration. Exp Eye Res 113: 172–181. S0014-4835(13)00138-3 [pii]; 10.1016/j.exer.2013.05.019 23791636

[21] Uranga RM, Katz S, Salvador GA (2013) Enhanced phosphatidylinositol 3-kinase (PI3K)/Akt signaling has pleiotropic targets in hippocampal neurons exposed to iron-induced oxidative stress. J Biol Chem 288: 19773–19784. M113.457622 [pii]; 10.1074/jbc.M113.457622 23687303 PMC3707681

[22] Uranga RM, Mateos MV, Giusto NM, Salvador GA (2007) Activation of phosphoinositide-3 kinase/Akt pathway by FeSO4 in rat cerebral cortex synaptic endings. J Neurosci Res 85: 2924–2932. 10.1002/jnr.21406 17600839

[23] Rouser G, Fkeischer S, Yamamoto A (1970) Two dimensional then layer chromatographic separation of polar lipids and determination of phospholipids by phosphorus analysis of spots. Lipids 5: 494–496. 5483450 10.1007/BF02531316

[24] BLIGH EG, DYER WJ (1959) A rapid method of total lipid extraction and purification. Can J Biochem Physiol 37: 911–917. 13671378 10.1139/o59-099

[25] Rodriguez DG, Uranga RM, Mateos MV, Giusto NM, Salvador GA (2012) Differential participation of phospholipase A2 isoforms during iron-induced retinal toxicity. Implications for age-related macular degeneration. Neurochem Int 61: 749–758. S0197-0186(12)00209-4 [pii]; 10.1016/j.neuint.2012.06.012 22732705

[26] Mateos MV, Uranga RM, Salvador GA, Giusto NM (2008) Activation of phosphatidylcholine signalling during oxidative stress in synaptic endings. Neurochem Int 53: 199–206. S0197-0186(08)00110-1 [pii]; 10.1016/j.neuint.2008.07.005 18692105

[27] Mateos MV, Giusto NM, Salvador GA (2012) Distinctive roles of PLD signaling elicited by oxidative stress in synaptic endings from adult and aged rats. Biochim Biophys Acta 1823: 2136–2148. S0167-4889(12)00262-5 [pii]; 10.1016/j.bbamcr.2012.09.005 23010583

[28] Ostrerova-Golts N, Petrucelli L, Hardy J, Lee JM, Farer M, Wolozin B (2000) The A53T alpha-synuclein mutation increases iron-dependent aggregation and toxicity. J Neurosci 20: 6048–6054. 20/16/6048 [pii]. 10934254 10.1523/JNEUROSCI.20-16-06048.2000 PMC6772599

[29] Mounsey RB, Teismann P (2012) Chelators in the treatment of iron accumulation in Parkinson's disease. Int J Cell Biol 2012: 983245 10.1155/2012/983245 22754573 PMC3382398

[30] Sian-Hulsmann J, Mandel S, Youdim MB, Riederer P (2011) The relevance of iron in the pathogenesis of Parkinson's disease. J Neurochem 118: 939–957. 10.1111/j.1471-4159.2010.07132.x 21138437

[31] Dusek P, Jankovic J, Le W (2012) Iron dysregulation in movement disorders. Neurobiol Dis 46: 1–18. S0969-9961(12)00009-5 [pii]; 10.1016/j.nbd.2011.12.054 22266337

[32] Stayte S, Vissel B (2014) Advances in non-dopaminergic treatments for Parkinson's disease. Front Neurosci 8: 113 10.3389/fnins.2014.00113 24904259 PMC4033125

[33] Glynn P (2013) Neuronal phospholipid deacylation is essential for axonal and synaptic integrity. Biochim Biophys Acta 1831: 633–641. S1388-1981(12)00158-8 [pii]; 10.1016/j.bbalip.2012.07.023 22903185

[34] Balboa MA, Balsinde J (2006) Oxidative stress and arachidonic acid mobilization. Biochim Biophys Acta 1761: 385–391. S1388-1981(06)00075-8 [pii]; 10.1016/j.bbalip.2006.03.014 16651022

[35] Bazan NG (2005) Lipid signaling in neural plasticity, brain repair, and neuroprotection. Mol Neurobiol 32: 89–103. MN:32:1:089 [pii]; 10.1385/MN:32:1:089 16077186

[36] Sun GY, Xu J, Jensen MD, Simonyi A (2004) Phospholipase A2 in the central nervous system: implications for neurodegenerative diseases. J Lipid Res 45: 205–213. 10.1194/jlr.R300016-JLR200;R300016-JLR200 [pii]. 14657205

[37] Else PL, Kraffe E (2014) Docosahexaenoic and arachidonic acid peroxidation: It's a within molecule cascade. Biochim Biophys Acta 1848: 417–421. S0005-2736(14)00374-5 [pii]; 10.1016/j.bbamem.2014.10.039 25450347

[38] Khaselev N, Murphy RC (1999) Susceptibility of plasmenyl glycerophosphoethanolamine lipids containing arachidonate to oxidative degradation. Free Radic Biol Med 26: 275–284. S0891-5849(98)00211-1 [pii]. 9895217 10.1016/s0891-5849(98)00211-1

[39] Hulbert AJ (2003) Life, death and membrane bilayers. J Exp Biol 206: 2303–2311. 12796449 10.1242/jeb.00399

[40] Ulmann L, Mimouni V, Roux S, Porsolt R, Poisson JP (2001) Brain and hippocampus fatty acid composition in phospholipid classes of aged-relative cognitive deficit rats. Prostaglandins Leukot Essent Fatty Acids 64: 189–195. S0952-3278(01)90260-1 [pii]; 10.1054/plef.2001.0260 11334555

[41] Shinzawa K, Sumi H, Ikawa M, Matsuoka Y, Okabe M, Sakoda S, et al (2008) Neuroaxonal dystrophy caused by group VIA phospholipase A2 deficiency in mice: a model of human neurodegenerative disease. J Neurosci 28: 2212–2220. 28/9/2212 [pii]; 10.1523/JNEUROSCI.4354-07.2008 18305254 PMC6671850

[42] Adibhatla RM, Hatcher JF, Dempsey RJ (2003) Phospholipase A2, hydroxyl radicals, and lipid peroxidation in transient cerebral ischemia. Antioxid Redox Signal 5: 647–654. 10.1089/152308603770310329 14580322

[43] Fonteh AN, Chiang J, Cipolla M, Hale J, Diallo F, Chirino A, et al (2013) Alterations in cerebrospinal fluid glycerophospholipids and phospholipase A2 activity in Alzheimer's disease. J Lipid Res 54: 2884–2897. jlr.M037622 [pii]; 10.1194/jlr.M037622 23868911 PMC3770101

[44] Wilson-Ashworth HA, Judd AM, Law RM, Freestone BD, Taylor S, Mizukawa MK, et al (2004) Formation of transient non-protein calcium pores by lysophospholipids in S49 Lymphoma cells. J Membr Biol 200: 25–33. 10.1007/s00232-004-0691-x 15386157

[45] Friedman JS, Chang B, Krauth DS, Lopez I, Waseem NH, Hurd RE, et al., (2010) Loss of lysophosphatidylcholine acyltransferase 1 leads to photoreceptor degeneration in rd11 mice. Proc Natl Acad Sci U S A 107: 15523–15528. 1002897107 [pii]; 10.1073/pnas.1002897107 20713727 PMC2932565

[46] He Q, Wang M, Petucci C, Gardell SJ, Han X (2013) Rotenone induces reductive stress and triacylglycerol deposition in C2C12 cells. Int J Biochem Cell Biol 45: 2749–2755. S1357-2725(13)00300-2 [pii]; 10.1016/j.biocel.2013.09.011 24104397 PMC3846609

